# Clinical implication of HLA class I expression in breast cancer

**DOI:** 10.1186/1471-2407-11-454

**Published:** 2011-10-20

**Authors:** Koichi Kaneko, Sumiya Ishigami, Yuko Kijima, Yawara Funasako, Munetsugu Hirata, Hiroshi Okumura, Hiroyuki Shinchi, Chihaya Koriyama, Shinichi Ueno, Heiji Yoshinaka, Shoji Natsugoe

**Affiliations:** 1 Department of Surgical Oncology, Breast and Endocrine Surgery, Kagoshima University School of Medicine, Kagoshima, Japan; 2 Department of Radiology, Graduate School of Medical and Dental Sciences, Kagoshima University, Kagoshima, Japan

**Keywords:** HLA class I, survival, T cell immunology, antitumor activity

## Abstract

**Background:**

Human leukocyte antigen (HLA)-class I molecules on tumor cells have been regarded as crucial sites where cytotoxic T lymphocytes (CTL) can recognize tumor-specific antigens and are strongly associated with anti-tumor activity. However, the clinical impact of HLA class I expression in breast cancer has not been clarified.

**Methods:**

A total of 212 breast cancer patients who received curative surgery from 1993 to 2003 were enrolled in the current study. HLA class I expression was examined immunohistochemically using an anti-HLA class I monoclonal antibody. The correlation between HLA class I positivity and clinical factors was analyzed.

**Results:**

The downregulation of HLA class I expression in breast cancer was observed in 69 patients (32.5%). HLA class I downregulation was significantly associated with nodal involvement (p < 0.05), TNM stage (p < 0.05), lymphatic invasion (p < 0.01), and venous invasion (p < 0.05). Patients with preserved HLA class I had significantly better disease-free interval (DFI) than those with loss of HLA class I (p < 0.05). However, in multivariable analysis, HLA class I was not selected as one of the independent prognostic factors of disease-free interval.

**Conclusion:**

The examination of HLA class I expression is useful for the prediction of tumor progression and recurrent risk of breast cancer via the antitumor immune system.

## Background

The antitumor activity via cytotoxic T lymphocytes (CTL) or tumor antigen has been clarified in the oncological field. Activation of anti-tumor CTL requires the recognition of immunogenic epitopes presented on various types of human leukocyte antigen (HLA) class I molecules on the tumor [1-4]. The concept of immune surveillance is maintaining the relationship between tumor-associated antigens (TAA) complexing with the HLA class I and tumor-specific cytotoxic T cells. These activated tumor-specific cytotoxic T cells can eliminate cancer cells specifically. The loss of HLA class I on the tumor is believed to lead to malfunction of recognition by the CD8+ T cells. It is already known that malignancies exhibit altered or lost expression of histocompatible antigens on the tumor cells [5-7]. Loss of HLA class I antigens appears to be a significant mechanism by which tumor cells escape specific immune attack and causes problems in the design of antitumor immunotherapy [7]. The loss of HLA class I antigens on tumor cells has been reported in several human tumors [5-7], and the loss of HLA class I molecules has been discussed in the context of tumor aggressiveness, such as differentiation of histology [8-10], invasiveness, and metastatic potential [5,11].

The non-covalent association with β 2-microglobulin (β 2 m) is essential for the structural stability and optimal function of HLA class I molecules [12]. Thus, several authors have used immunostaining of β 2 m for the analysis of overall surface expression of HLA class I molecules [9,11,13]. However, there are often difficulties in evaluating immunostaining using anti-MHC class I monoclonal antibodies (mAbs), such as W6/32, HC-10, or HC-A2, as these type of antibodies are not ideal for the immunostaining of formalin-fixed, paraffin-embedded tissue. Moreover, these antibodies are not fully recognized whole HLA class I properly. Recently, EMR8-5, a monoclonal antibody against HLA class I heavy chains (HLA-A, B, C), has been validated in HLA class I immunohistochemistry [14-16], and used to investigate HLA class I expression in osteosarcoma [14], non-small cell lung cancer [15], and renal cell carcinoma [16]. The clinical implication of the HLA class I expression has been discussed and reviewed in breast cancer and it is not clarified [17-21]. Moreover, there have been no studies on HLA class I expression of breast cancer by EMR8-5. The present study assessed HLA class I expression in invasive breast cancer by immunohistochemistry using the EMR8-5 antibody, analyzed associations with clinicopathological factors, and discussed the clinical implication of HLA class I-positive breast cancer.

## Methods

### Patients

A total of 212 breast cancer patients, who consecutively underwent curative operation for primary invasive breast cancer at Kagoshima University Hospital from 1993 and 2003, were enrolled in the current study. None of the patients received any preoperative systemic chemotherapy or endocrine therapy. Clinicopathological features were documented according to TNM classification [17]. All patients were female and their mean age was 56 (ranging from age 23 to 90). One hundred and fifty-three underwent mastectomy and the remaining 59 underwent partial resection. A total of 84 patients had nodal involvement, and the numbers of patients with stages I, II, and III were 70, 101, and 41, respectively. This study was approved by the Ethical Committee of the University of Kagoshima, and written informed consent was obtained from all individuals. Overexpression of the estrogen receptor (ER), progesterone receptor (PgR), and HER2 were examined by immunohistochemical staining using the appropriate primary antibodies. Distinct staining of the nucleus in more than 10% of tumor cells was recorded as positive for ER and PgR, and strong membrane staining in more than 10% of invasive lesions was recorded as positive for HER2.

### Immunohistochemical analysis of HLA class I in breast cancer

HLA class I expression was investigated by immunohistochemical staining with the monoclonal anti-pan HLA-class I antibody 5 EMR8-5 (Cosmo Bio Co., Tokyo, Japan). EMR8-5 is an anti-pan HLA class I monoclonal antibody, which can recognize all of HLA A, B, and C heavy chain even in formalin-fixed tissue [22].

The avidin-biotin complex (ABC) method was used to visualize HLA class I expression in breast cancer. Human tonsil sections were used as positive controls for HLA class I, and in the negative controls, the primary antibody was replaced with buffer. The ABC method was performed in accordance with previous reports [14-16]. Namely, 4 μm paraffin-embedded sections of breast cancer were de-paraffinized and soaked in PBS. The sections were treated with 3% H_2_O_2 _for 30 min in order to block endogenous tissue peroxidases, followed by treatment with rabbit serum for 60 min in order to reduce non-specific binding. Primary anti-HLA-class I antibody was diluted to 1:100 and incubated with the tonsil sections at 4°C overnight. Sections were rinsed in PBS and visualized using standard techniques for labeled avidin-biotin immunoperoxidase staining.

All specimens were reviewed independently using light microscopy for at least five areas at a 400 × magnification by two investigators (KK and SI) who were blinded with respect to the clinicopathological data. The intensity of HLA-class I staining was evaluated in accordance with a previous report [23], using the following criteria: strongly positive (positive), defined as complete membrane staining in 40% or more of tumor cells; weakly positive (negative), any lesser degree of staining appreciable in tumor cells; and absent (negative), no appreciable staining in tumor cells.

### Survival analysis and statistical evaluation

Postoperative intervals were estimated by the Kaplan-Meier method. In the survival analysis, we excluded the cases that had not undergone curative resection. The endpoint of survival analysis was defined as the day of death of each patient from not only cancer-related events but also other causes. That of disease-free survival (DFS) was defined as a locoregional recurrence or distant metastasis of breast cancer in soft tissue, lymph nodes, liver, lung, brain, and/or bone by physical and/or pathological examination. Overall survival (OS) and DFS of 212 patients were 88.4% and 83.0%, respectively. Median and mean survivals of these patients were 71.9 and 76.2 months, respectively.

Significant differences of DFS and OS were calculated using the log-rank test, and significant differences in categorical variables were analyzed by the χ^2^-test. Univariate and multivariable analyses of the postoperative outcome were conducted using Cox's proportional hazards model. Differences were considered significant at p < 0.05. All statistical analyses were performed using Stat View 5.0 software.

## Results

### Expression and evaluation of HLA class I in breast cancer tissue

**Figure 1:**
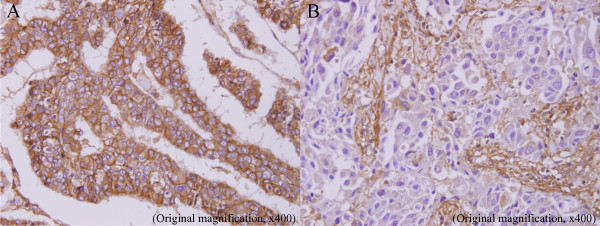
**Representative images of immunostaining of HLA class I in invasive breast cancer**. A: positive expression, B: downregulated expression.

HLA class I positivity was found in not only the membrane of tumor cells but also in the cytoplasm of tumor cells (Figure 1). In addition, Some stromal lymphocytes also showed HLA class I positivity. Generally, there was little HLA class I positivity in normal mammary glands adjacent to cancerous tissue. According to the previously mentioned evaluation, 69 patients (32.5%) had strong HLA class I expression, 64 patients (30.2%) had weak expression, and the remaining 79 patients (37.3%) lacked expression. Patients with weak or no expression of HLA class I antigens in breast cancer were classified as the downregulated HLA class I group. In contrast, patients with more than 40% of HLA class I positivity were classified as the positive HLA class I group.

### Association between HLA class I and clinicopathological factors

The downregulation of HLA class I expression in breast cancer was significantly associated with nodal metastasis, TNM, lymphatic invasion, and venous invasion (p = 0.04, p = 0.01, p = 0.006, and p = 0.04, respectively) (Table 1). There was no significant association between HLA class I expression and histology or hormonal status.

**Table 1:** Association between clinical factors and HLA class I expression in 212 breast cancer patients

	Positive (n = 69)	Negative (n = 143)	p value
Age (years)			
< 50	23 (11%)	59 (28%)	0.2669
51 <	46 (22%)	84 (40%)	
Tumor size			
T1	35 (17%)	51 (24%)	0.0641
T2	31 (15%)	72 (34%)	
T3	1	12 (57%)	
T4	2	8 (4%)	
Nodal invasion			
Negative	49 (23%)	79 (37%)	***0.0278***
Positive	20 (9%)	64 (30%)	
Estrogen receptor			
Negative	18 (8%)	53 (25%)	0.1126
Positive	51 (24%)	90 (42%)	
Progesterone receptor			
Negative	25 (12%)	64 (30%)	0.2387
Positive	44 (21%)	79 (37%)	
Lymphatic invasion			
Negative	46 (22%)	67 (32%)	***0.0067***
Positive	23 (11%)	76 (36%)	
Venous invation			
Negative	66 (31%)	122 (58%)	***0.0260***
Positive	3 (1%)	21 (10%)	
TNM			
I	31 (15%)	39 (18%)	***0.0110***
II	31 (15%)	70 (33%)	
III	7 (3%)	34 (16%)	
Histology			
IDC	67 (32%)	142 (67%)	0.2040
ILC	2	1	
HER2 receptor			
Negative	63 (30%)	123 (58%)	0.4539
Positive	6 (3%)	17 (8%)	

Patients' overall survival (OS) and disease-free survival (DFS) with or without HLA class I expression in breast cancer

**Figure 2:**
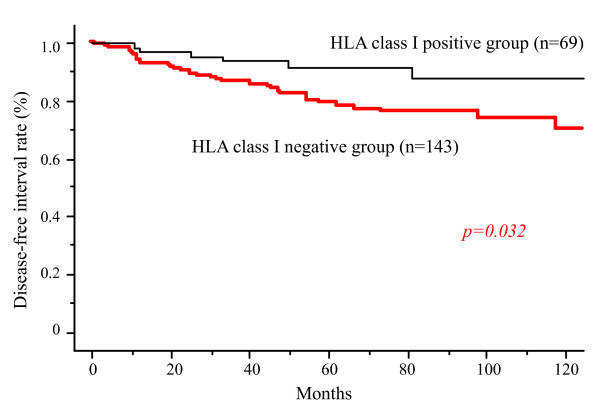
**Disease free survival according to HLA class I expression**. Surgical outcome of the HLA class I positive group was significantly better than that of the downregulated group (p < 0.05).

Postoperative OS was not significantly different according to HLA class I expression. However, patients with HLA class I positivity had significantly longer DFS than those without HLA class I positivity (p < 0.05) (Figure 2). Using univariate analysis, seven clinical factors, including HLA class I expression, were selected as significant for DFS (Table 2). According to multivariable analysis using these seven factors, lymph node metastasis, progesterone receptor, and vascular invasion were independent prognostic factors. HLA class I expression was not selected as an independent factor for DFS (Table 2).

**Table 2:** Univariate and multivariable analyses for DFS

		Univariate analysis		Multivariable analysis	
		
		Disease free interval		Disease free interval	
**Variables**	**Categories**	**HR (95% confidence interval)**	**p value**	**HR (95% confidence interval)**	**p value**

Tumor size	T3+T4 (vs T1+T2)	0.249 (0.120 - 0.514)	***P = 0.0020***	0.459 (0.212 - 0.995)	***P = 0.0485***
Nodal involvement	N1 (vs N0)	0.148 (0.068 - 0.324)	***P < 0.0001***	0.230 (0.092 - 0.574)	***P = 0.0016***
Estrogen receptor	Positive (vs negative)	1.656 (0.872 - 3.146)	0.1231		
Progesterone receptor	Positive (vs negative)	1.932 (1.019 - 3.664)	***P = 0.0437***	2.168 (1.109 - 4.240)	***P = 0.0237***
Lymphatic invasion	Positive (vs negative)	0.246 (0.117 - 0.521)	***P = 0.0020***	0.871 (0.346 - 2.194)	0.7689
Vascular invasion	Positive (vs negative)	0.184 (0.094 - 0.361)	***P < 0.0001***	0.423 (0.198 - 0.904)	***P = 0.0264***
HER2 receptor	Positive (vs negative)	0.653 (0.272 - 1.565)	0.3390		
HLA class I expression	Negative (vs positive)	2.513 (1.050 - 6.012)	***P = 0.0384***	1.691 (0.696 - 4.110)	0.2462

## Discussion

The monoclonal antibody EMR8-5 can recognize all of HLA A, B, and C heavy chain even in formalin-fixed tissue. In this context, EMR8-5 can recognize whole HLA molecules, and its validity was supported by the immunostaining performed in our current studies. Cordon et al. reported that HLA class I positivity examined using the conventional HLA class I antibody W6/32, which also recognizes all HLA class I antigens, was 30% of HLA class I positivity in breast cancer, similar to that shown in our present study [24]. In contrast, Madjd et al. investigated HLA class I expression in breast cancer using a HC-10 antibody [24], and demonstrated that HLA class I negativity correlated with a better postoperative outcome. These results conflicted with the data in our studies. This discrepancy may be explained by the fact that whereas the HC10 mAb scarcely reacts with HLA-A alleles, the anti- HLA class I heavy chain mAb EMR8-5 can detect all recombinant proteins of HLA-A, B, and C alleles by immunoblot analysis [25]. In addition, EMR8-5 can be applied to paraffin-fixed specimens, so in this context it is an ideal antibody for evaluating cancerous HLA class I antigen expression.

In our current study, the downregulation of HLA class I expression in breast cancer was 66%, which was more than that in gastric (32%) [22] and esophageal cancer (43%) [26], and osteosarcoma (55%) [14], but less than the downregulation in lung cancer (70%) [15]. The degree of HLA class I loss may be affected by organ specificity. For example, Ishigami et al. speculated that highly preserved HLA class I expression in gastric cancer is partly due to exogenous stimulation from gastritis or bacterial infection of Helicobacter pylori [22]. The differences in HLA class I expression in breast cancer may also be explained by the possible inflammation and proteolysis that can occur at the sites of breast cancer origin. These are important steps linked both to HLA loss and cancer aggressiveness. In this study, there was little HLA class I positivity in normal mammary gland tissue. In contrast, HLA class I antigens were preserved in early breast cancer, and cancerous HLA class I antigens were newly expressed or reduced according to the tumor extension. Although T1 tumors had 41% HLA class I positivity, T3-4 tumors only showed 15% positivity. According to tumor extension, preservation of HLA class I of the tumor was reduced. This clinical trait was also reported in other types of malignancies, such as gastrointestinal cancer [22,26] and sarcoma [14]. It is possible that, in the process of tumor extension, tumors that lost HLA class I survived and escaped from antigen-specific CTL-mediated lysis leading to tumor dissemination and metastasis. However, these results do not fully explain the relationship with metastasis, therefore we need to perform more analyses comparing the results among in situ, lobular and ductal breast cancers on key parameters, such as VEGF, MMP etc.

In the current study, the downregulation of HLA class I expression was significantly associated with lymphatic and nodal invasion. Mizukami et al. showed that when HLA class I-positive esophageal cancer metastasized to the lymph node, tumor cells completely lost HLA class I expression in this system [22]. Zia et al. investigated the immunological characteristics of isolated cancer cells (ITC) in bone marrow and found that ITCs with the HLA class I downregulation phenotype were often derived from poorly differentiated primary breast carcinomas which was associated with a short survival period in breast cancer [27]. Therefore, cancerous HLA class I downregulation seems to be conducive to metastasis to other organs.

Patients with positive HLA class I expression showed a better DFS in comparison with those with downregulation of HLA class I expression. This result differs from that for esophageal cancer [26]. In breast cancer, the average OS is generally better than those in other malignancies; DFS is often used to evaluate aggressiveness of biological markers in breast cancer. In this context, significant differentiation in DFS seems to be meaningful.

It has been clarified that HLA molecule inactivity depends not only on the expression of HLA class I molecules themselves, but also on the post-transcriptional course that mainly affects β2-microglobulin gene expression. Aptsiauri et al. [28] showed that if apparent tumor cells expressed HLA class I, various types of HLA class I alterations were found in malignancies and in the molecular mechanisms that underlie these defects. In this context, the HLA class I molecules preserved in these breast cancers may exhibit altered expression and dysfunction as antigen presentation molecules. It seems to be difficult to precisely evaluate HLA class I expression, however, in the current study, we evaluate HLA expression including in β2 microglobulin expression using the EMR8-5 antibody.

## Conclusions

The downregulation of HLA class I expression frequently occurred in breast cancer, in a similar manner to what has been seen in several other cancers, and it may also be associated with tumor progression and relapse. Therefore HLA class I status may be useful with other well-known prognostic factors like nodal involvement and hormone status to evaluate postoperative outcomes in breast cancer.

## Abbreviations

ABC method: Avidin-biotin complex method; CTL: cytotoxic T lymphocytes; DFS: disease free survival; HLA: Human leukocyte antigen; OS: overall survival.

## Competing interests

The authors declare that they have no competing interests.

## Authors' contributions

KY carried out the immuno histopathological studies and performed the statistical analysis. SI participated in its design and coordination. All authors read and approved the final manuscript.

## Pre-publication history

The pre-publication history for this paper can be accessed here:

http://www.biomedcentral.com/1471-2407/11/454/prepub
